# Joint-GWAS, Linkage Mapping, and Transcriptome Analysis to Reveal the Genetic Basis of Plant Architecture-Related Traits in Maize

**DOI:** 10.3390/ijms25052694

**Published:** 2024-02-26

**Authors:** Xuefeng Lu, Pengfei Liu, Liang Tu, Xiangyang Guo, Angui Wang, Yunfang Zhu, Yulin Jiang, Chunlan Zhang, Yan Xu, Zehui Chen, Xun Wu

**Affiliations:** 1 Institute of Upland Food Crops, Guizhou Academy of Agricultural Sciences, Guiyang 550006, China; lxf0403791@126.com (X.L.);; 2 Ministry of Agriculture and Rural Affairs Key Laboratory of Crop Genetic Resources and Germplasm Innovation in Karst Region, Guiyang 550006, China

**Keywords:** maize (*Zea mays* L.), plant architecture, linkage mapping, QTL, candidate gene

## Abstract

Plant architecture is one of the key factors affecting maize yield formation and can be divided into secondary traits, such as plant height (PH), ear height (EH), and leaf number (LN). It is a viable approach for exploiting genetic resources to improve plant density. In this study, one natural panel of 226 inbred lines and 150 family lines derived from the offspring of T32 crossed with Qi319 were genotyped by using the MaizeSNP50 chip and the genotyping by sequence (GBS) method and phenotyped under three different environments. Based on the results, a genome-wide association study (GWAS) and linkage mapping were analyzed by using the MLM and ICIM models, respectively. The results showed that 120 QTNs (quantitative trait nucleotides) and 32 QTL (quantitative trait loci) related to plant architecture were identified, including four QTL and 40 QTNs of PH, eight QTL and 41 QTNs of EH, and 20 QTL and 39 QTNs of LN. One dominant QTL, qLN7-2, was identified in the Zhangye environment. Six QTNs were commonly identified to be related to PH, EH, and LN in different environments. The candidate gene analysis revealed that *Zm00001d021574* was involved in regulating plant architecture traits through the autophagy pathway, and *Zm00001d044730* was predicted to interact with the male sterility-related gene ms26. These results provide abundant genetic resources for improving maize plant architecture traits by using approaches to biological breeding.

## 1. Introduction

Maize is an important food and feed resource, and its yield is impacted by single plant yield and planting density per unit area. Excessive use of pesticides and fertilizers can lead to soil contamination, so exploiting genetic resources is a significant approach to improving maize production. Ideal architecture-related traits play a key role in increasing the planting density per unit area and maize yield. Reports have shown that maize plant architecture-related traits are divided into four secondary traits: plant height (PH), ear height (EH), leaf number (LN), and leaf angle (LA) [1,2]. The ideal PH, EH, and LN could increase the utilization rate of light energy and improve plant photosynthesis to increase maize yield [2,3,4], which has been the focus of breeders. Maize plant architecture is a complex quantitative trait subject to a combination of environmental and genetic effects. QTL mapping is a common research approach for quantitative traits. Over the decades, many plant architecture-related QTL have been identified and used for molecular marker-assisted breeding, which has provided some evidence for the exploration of the genetic and molecular mechanisms for plant architecture variation.

Many QTL were detected for plant architecture in different environments using various materials, including the F2:3 population, RILs (recombinant inbred lines), etc. Zheng et al. [5] used one RIL population derived from Mo17 and Huangzao4 to detect QTL related to plant architecture under two nitrogen conditions. Eight QTL were detected in a high nitrogen treatment, including one for PH, three for EH, and four for LN, and ten QTL were detected under low nitrogen treatment, including one for PH, five for EH, and four for LN. These QTL were distributed on chromosomes one, two, three, seven, and nine [5]. Based on a high-density genetic map constructed using 4183 bin markers via the GBS analysis of 314 RILs derived from the inbred lines of Ye478 and Qi319, 35 QTL affecting plant architecture were detected in three environments, including 14 for PH and 14 for EH [6]. Using the GBS method, Wang et al. found 51 QTL related to plant architecture, with eight QTL for PH and nine QTL for EH [7]. Using the SLAF-seq method, Fei identified 11 and 13 QTL associated with PH and EH, respectively. Among these results, one candidate gene, *Zm00001d042292*, was identified to be involved in the auxin signaling pathway and is located in a QTL of plant height and ear height on chromosome three [8]. Li et al. reported a major-effect locus related to the number of leaves above the primary ear by using a large set of 866 maize-teosinte BC2S3 RILs genotyped by using 19,838 single-nucleotide polymorphism markers [2]. Cui et al. used an F2:3 population including 192 family lines, one natural population with 437 inbred lines, and a pair of near-isogenic lines to identify two major QTL, qLA3-4 and qLA7-1. According to these results, one candidate gene, *lfy1*, related to LA was cloned [9].

Genome-wide association studies (GWASs) have become a widely accepted strategy to identify loci linked to agronomic traits and stress resistance in maize, such as plant height [1,10], male inflorescence size [11], major ear [12], drought tolerance [13], etc. By using three methods (i.e., separate linkage mapping (SLM), joint linkage mapping (JLM), and GWASs) to analyze maize advanced backcross-nested association mapping populations consisting of 1625 BC1F4/BC2F4 lines, Zhao identified 9–11 QTL associated with PH and 6–8 QTL associated with EH [14]. Ruanjaichon et al. identified 12 significantly associated SNPs on chromosomes 3, 4, 5, and 7 via a GWAS in an association panel consisting of 250 sweet corn, waxy corn inbred, and RILs; a candidate gene, *sh2*, affecting starch metabolism in the maize endosperm. In simple words, the GWAS has been applied successfully as a powerful tool to the crucial traits. An increasing number of candidate genes related to plant architecture was detected in a previous study. Du et al. finely mapped *lfy1* into one 55 kb interval and found two candidate genes, *GRMZM2G072052* and *GRMZM2G072080*, and the polymorphism markers on the two candidate genes were all cosegregated with the leafy phenotype [15]. *ZCN8* was identified as a candidate gene for leaf number [16]. Li et al. found that ACC (1-aminocyclopropane-1-carboxylic acid) and ethylene content significantly increased in the maize mutant semidwarf3 (*sdw3*), resulting in shorter plant height and an increased leaf angle. In addition, an important pleiotropy candidate gene, *ZmACS7*, was cloned by using QTL mapping and was found to play an important role in LN formation during maize growth [17]. Different alleles of *ZmPGP1* were found to be related to PH, EH, LA, ear length (EL), yield, and root development under aluminum stress [18,19,20,21,22,23]. The resequencing of *ZmPGP1* in different materials revealed that this gene may be selected during domestication and modification, and the significant variation could be used to develop functional markers to improve maize plant structure and ear traits [24]. *ZmBZR1* appears to be the main regulator of maize plant height [25]. Wei et al. reported that *ZmSPL* genes play an important role in regulating plant architecture traits, including plant/ear height and leaf angle. [26]. These reports have provided much more useful information for the improvement of ideal maize plant architecture. However, different genetic loci were identified in different reports, which may be caused by the differences in the detection methods, materials, and marker types/marker numbers used in the reports.

In our study, one natural panel of 226 inbred lines and 150 family lines derived from the offspring of T32 crossed with Qi319 were genotyped by using the MaizeSNP50 chip and the genotyping by sequence (GBS) method. These two populations were also phenotyped under three different environments: Sanya, Guiyang, and Zhangye. The objectives were to (1) identify the important QTL/QTNs related to plant architecture, (2) explore the ideal plant architecture-related candidate genes and their functions, and (3) analyze the regulatory pathways of some of the candidate genes. These results not only provide abundant genetic resources for improving maize plant architecture traits but also provide target regions for the further fine mapping of maize plant architecture traits.

## 2. Results

### 2.1. Phenotypic Analysis of the Plant Architecture-Related Traits

**Figure 1:**
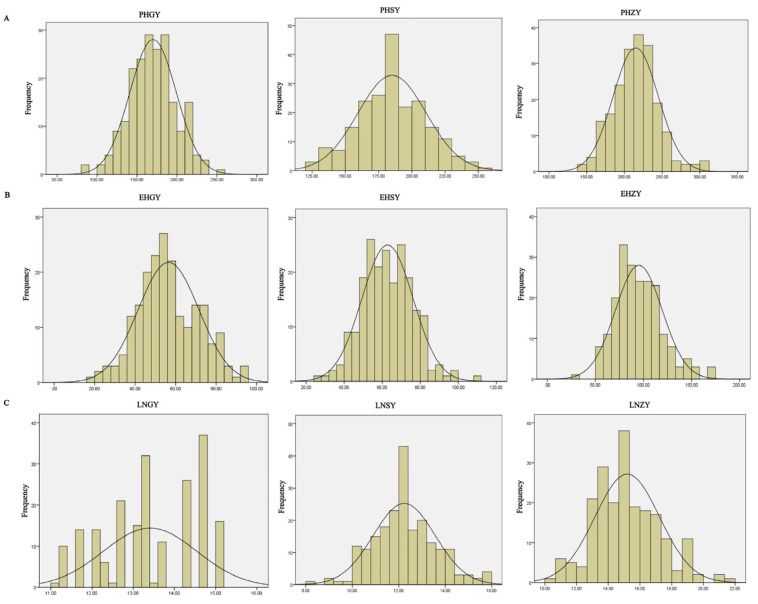
The frequency distribution of plant architecture-related traits. (**A**) Frequency distribution of PH in Guiyang, Sanya, and Zhangye. (**B**) Frequency distribution of EH in Guiyang, Sanya, and Zhangye. (**C**) Frequency distribution of LN in Guiyang, Sanya, and Zhangye. GY, SY, and ZY represent Guiyang, Sanya, and Zhangye, respectively.

**Figure 2:**
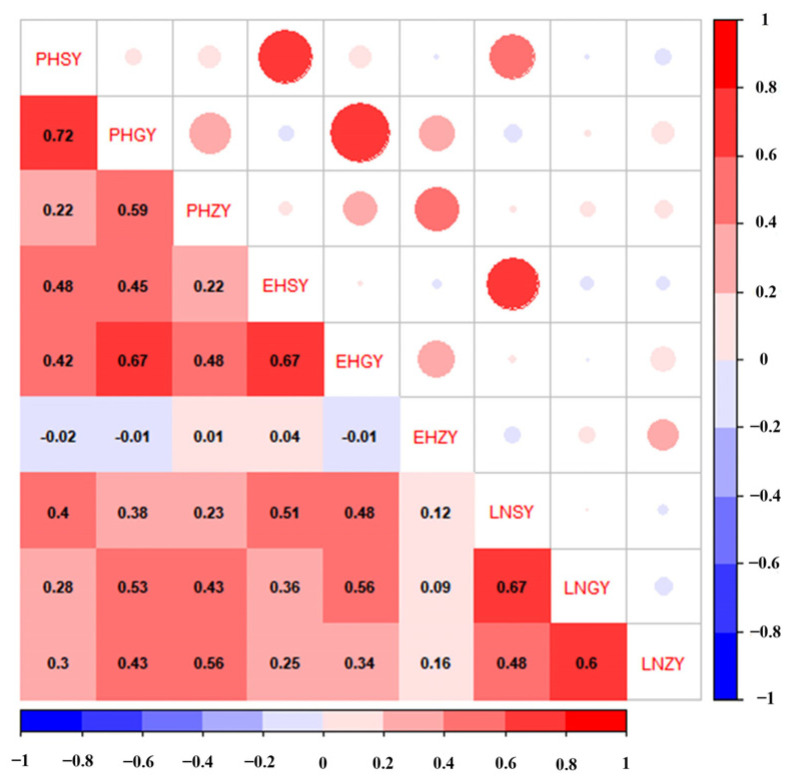
Diagram of the correlation analysis between the paired plant architecture-related traits in different environments. The positive values are shown in red, while the negative correlations are shown in blue.

The descriptive analysis showed that the PH varied from 138.33 cm to 310.00 cm in Zhangye, from 82.33 cm to 259.67 cm in Guiyang, and from 123.33 cm to 258.00 cm in Sanya. The EH varied from 31.67 cm to 171.67 cm in Zhangye, from 19.67 cm to 94.67 cm in Guiyang, and from 26.67 cm to 108.33 cm in Sanya. The LN varied from 10.00 to 21.67 in Zhangye, from 11.00 to 15.00 in Guiyang, and from 8.33 to 15.67 in Sanya. The absolute values of skewness and kurtosis were less than one (Figure 1). The variation coefficient of LN in Guiyang was less than 10%, but the variation coefficients of plant height and ear height in the three environments and leaf number in Sanya and Zhangye were greater than 10%, which indicated abundant phenotypic variation in the plant architecture (Table 1). The variance analysis indicated that the PH, EH, and LN showed significant differences among the different inbred lines (Table 2). The correlation analysis of the plant architecture parameters in Zhangye, Guiyang, and Sanya showed that ear height in Zhangye (EHZY) was negatively correlated with plant height in Sanya (PHSY), plant height in Guiyang (PHGY), and ear height in Guiyang (EHGY), while the other traits were positively correlated with each other (Figure 2).

### 2.2. Genome-Wide Association Study

**Figure 3:**
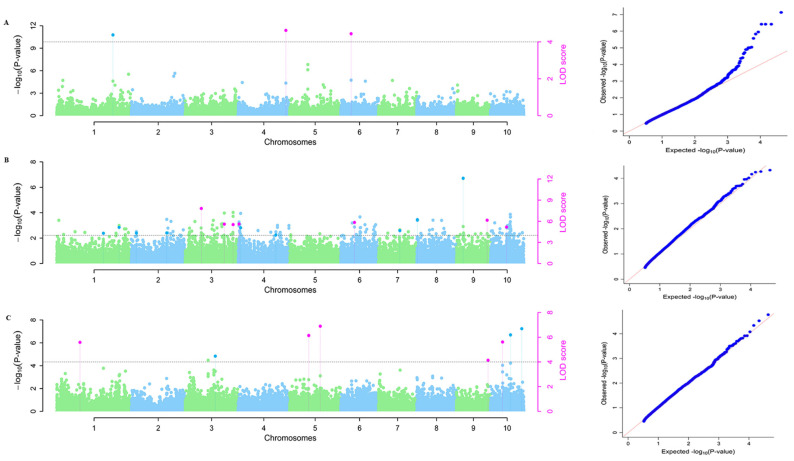
Manhattan and Q–Q plots for maize plant height traits under different environments. (**A**) Guiyang, (**B**) Zhangye, (**C**) Sanya. A significant LOD score threshold level was more than 3.0. The left plots are Manhattan, and the right plots are quantile-quantile (Q–Q).

For plant height, 22, 3, and 15 significant QTNs in the Zhangye, Guiyang, and Sanya environments were identified, respectively, which explained 1.75% to 25.87% of the genetic variation. For ear height, 18, 16, and 7 QTNs in the Zhangye, Guiyang, and Sanya environments, respectively, were identified, which explained 1.65% to 36.18% of the genetic variation. For leaf number, 16, 16, and 7 QTNs in the Zhangye, Guiyang, and Sanya environments, respectively, were identified, which explained 2.21% to 19.21% of the genetic variation (Table S1, Figure 3, Figures S1 and S2). Interestingly, six QTNs were commonly identified to be related to PH, EH, and LN in the different environments. The linked markers were PZE-106027247 (PHGY and EHGY), PZE-101198702 (PHSY and EHGY), SYN21465 (EHZY and LNZY), SYN34204 (EHZY, LNZY, and LNGY), SYN37324 (EHGY and LNGY), and PUT-163a-78121249-4396 (EHGY and LNGY); the markers are listed in Table 3.

Six markers were searched in the Maize GDB database, which revealed that only two SNPs among them, SYN34204 and SYN37324, were associated with two genes, i.e., *Zm00001d021574* and *Zm00001d044730*, respectively. SYN34204 was identified to be correlated with ear height and leaf number in Zhangye and leaf number in Guiyang, and SYN37324 was detected to be correlated with ear height and leaf number only in Guiyang. The gene annotation in the NCBI (National Center for Biotechnology Information) shows that *Zm00001d021574* encodes laz1-1 and *Zm00001d044730* encodes the calcium-binding EF-hand protein.

### 2.3. Linkage Analysis of the Plant Architecture-Related Traits

**Figure 4:**
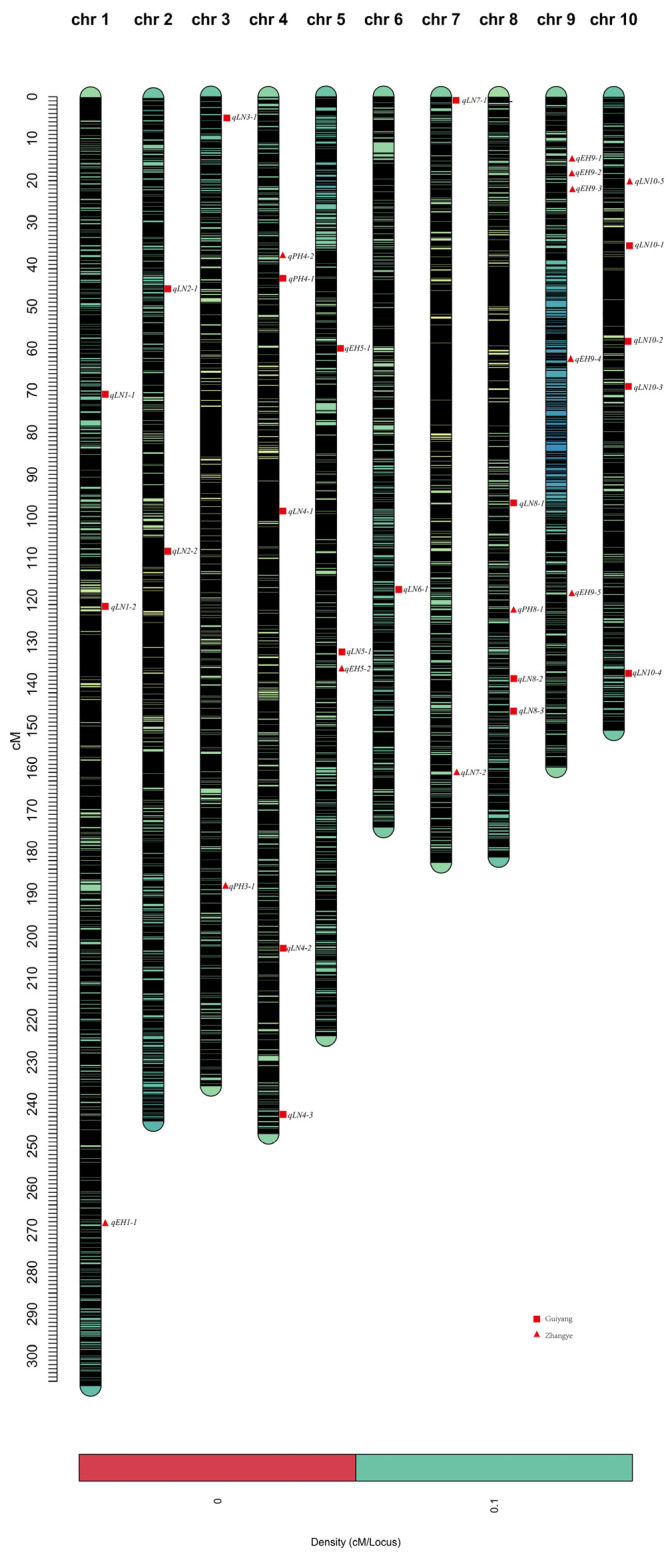
Distribution of QTL on the genetic map. The squares represent the QTL detected in Guiyang, and the triangles represent the QTL detected in Zhangye.

In the linkage analysis, a total of 32 QTL were detected, with 4 QTL related to PH, distributed at chromosome 3, chromosome 4 and chromosome 8, explaining 3.64~6.57% of the variation; 8 QTL related to EH, distributed at chromosome 1, chromosome 5 and chromosome 9, explaining 3.94~9.20% of the variation; and 20 QTL related to LN, distributed at chromosome 1 to chromosome 10, except for chromosome 9, explaining 1.66~18.11% of the variation (Table 4, Figure 4). One QTL, qLN7-2, located on chromosome 7, explained more than 10% of the phenotypic variance and could be a major QTL, with a positive additive effect derived from the female parent of Qi319.

### 2.4. Transcriptome Analysis

**Figure 5:**
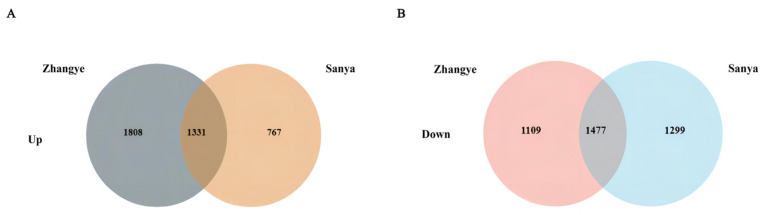
DEGs identified between T32 and Qi319 in the different environments of Zhangye and Sanya. (**A**) Upregulated common DEGs in different environments. (**B**) Downregulated common DEGs in different environments.

**Figure 6:**
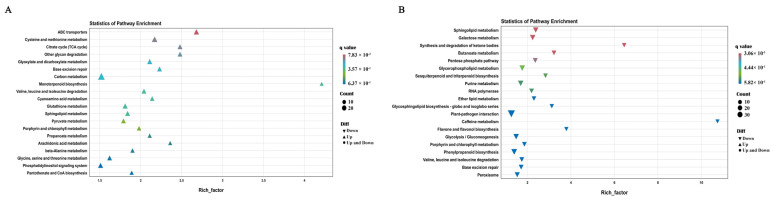
KEGG pathway-rich distribution map of the differentially expressed genes. (**A**) Pathway enrichment of the upregulated genes. (**B**) Pathway enrichment of the downregulated genes.

**Figure 7:**
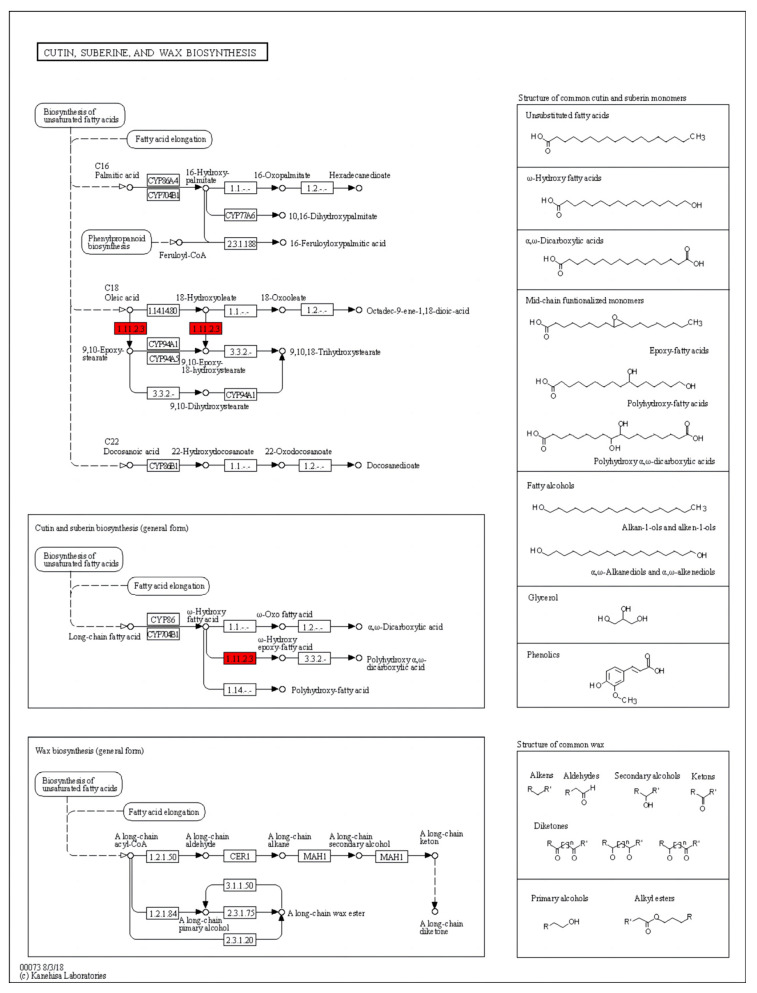
KEGG pathway map of *Zm00001d044730*.

To identify the genes involved in the plant architecture traits, the differentially expressed genes (DEGs) between T32 and Qi319 in different environments (Sanya and Zhangye) were identified. In the Sanya environment, 4874 genes showed differential expression, of which 2098 genes were significantly upregulated, and 2776 genes were significantly downregulated expression in T32 when compared to Qi319. A total of 5725 genes showed differential expression in the Zhangye environment, of which 3139 genes were significantly upregulated, and 2586 were significantly downregulated in T32 when compared to Qi319. Among these DEGs, 1331 genes were upregulated, and 1477 genes were downregulated in both environments (Figure 5). To identify the regulatory pathway, the KEGG pathway analysis of common upregulated and downregulated genes in both environments was conducted. It showed that ABC transporter, cysteine, and methionine metabolism were the most significantly enriched pathways, which included 14 and 16 upregulated genes, respectively (Figure 6A). Sphingolipid metabolism and galactose metabolism were the most significantly enriched pathways, which included 13 and 12 downregulated genes, respectively (Figure 6B). Interestingly, five downregulated genes were identified in both pathways (Table 5). To identify the regulatory pathway involved in the two candidate genes, *Zm00001d021574* and *Zm00001d044730*, we searched the two genes in the DEGs and checked the gene annotations. It showed that the candidate gene *Zm00001d044730* coding caleosin-related protein was a significantly upregulated gene in Zhangye and Sanya and participated in the biosynthesis of cutin, suberin, and wax, which are involved in the regulation of plant architecture formation (Figure 7). In the DEGs, *Zm00001d021574* was not detected in both environments.

### 2.5. Function Prediction of the Candidate Genes

**Figure 8:**
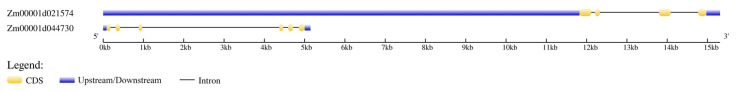
The gene structure of two candidate genes.

**Figure 9:**
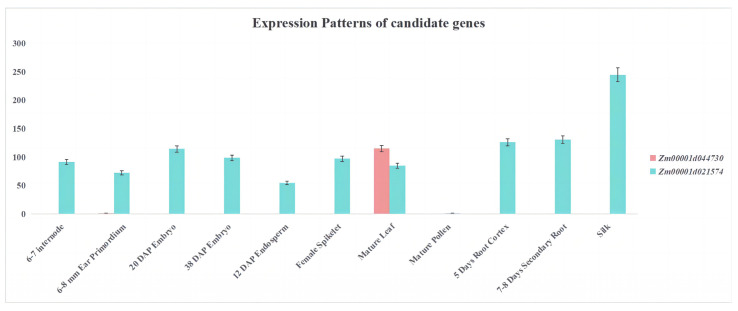
Analysis of the expression patterns of two candidate genes.

**Figure 10:**
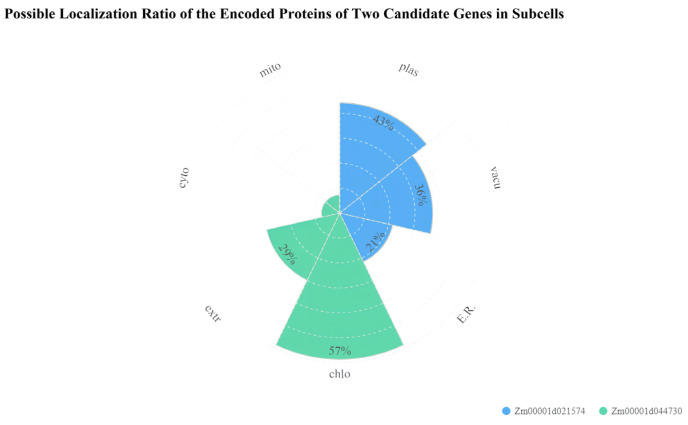
Possible localization ratio of the encoded proteins of two candidate genes in subcells. Plas: plasma membrane, Vacu: vacuolar membrane, E.R.: endoplasmic reticulum, Chlo: chloroplast, Extr: extracellular, Cyto: cytosol, Mito: mitochondrion.

**Figure 11:**
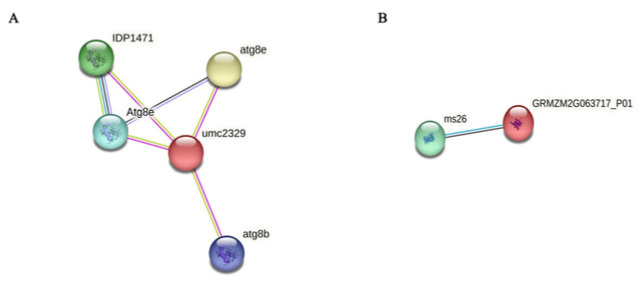
Interaction analysis of two candidate genes. The nodes represent the genes, and the lines represent the interactions between the genes. (**A**) Umc2329 is the *Zm00001d021574* gene. (**B**) GRMZM2G06371_P01 is the *Zm00001d044730* gene.

The gene structure of the two candidate genes identified in this paper showed that both genes had upstream and downstream regulatory regions. The CDS (coding sequence) of *Zm00001d021574* was 909 bp with three introns. The CDS of *Zm00001d044730* was 669 bp with five introns (Figure 8). The temporal and spatial expression pattern of *Zm00001d021574* showed that it was expressed in all tissues, but the expression level was highest in the silk and the lowest in mature pollen. *Zm00001d044730* was only expressed in 6–8 mm ear primordia and mature leaves; the lowest expression was found in 6–8 mm ear primordia, and the highest expression was found in mature leaves (Figure 9). Subcellular localization predicted that the LAZ1-1 protein encoded by Zm00001d021574 is expressed in the plasma membrane, and the calcium-binding EF-hand protein encoded by *Zm00001d044730* is expressed in chloroplasts (Figure 10). The protein interaction analysis showed that IDP1471, Atg8e, atg8b, and atg8e interacted with *Zm00001d021574* (umc2329) (Figure 11A). Gene annotation in the NCBI database showed that all four genes belonged to the Atg8 protein family. The *Zm00001d021574* gene contains an organic solute transporter conserved domain, which may be involved in the autophagy pathway with the four Atg8 family proteins. The interaction between the *Zm00001d044730* gene and the *ms26* gene was found in the STRING database (Figure 11B). The *Zm00001d044730* gene contains the caleosin protein conserved domain, and *ms26* is a cytochrome P450-like gene and may have other functions in addition to its involvement in male sterility.

## 3. Discussion

Ideal plant architecture can increase the planting density and harvest index to increase maize yield. The PH, EH, and LN are important secondary factors that can affect plant architecture. In the recent study, different materials and methods selected to conduct the study could generate variable results. T32 was a foundation parental line derived from the Suwan germplasm, which showed a high combining ability, and Qi319 was another foundation line derived from temperate maize germplasm in tropical regions. These two lines showed significant discrepancies in plant architecture traits in different environments. Therefore, plant architecture traits are not only regulated by genotype but also easily affected by the environment. In a previous study, the interaction effect of genotype and the environment on maize plant architecture-related traits was significant [8]. Revealing the genetic basis of plant architecture traits and identifying candidate genes will probably improve maize yields in different environments.

GWASs have been widely applied to parsing associations with genes and phenotypes in maize plant architecture traits. The GWAS identified 41 SNPs for PH and EH, and the combined QTL analysis detected a likely candidate gene, *C2H2*, the zinc finger family protein, in the positioning interval [10]. The maize doubled haploid lines derived from 52 exotic maize races used to conduct the GWAS analysis later identified twelve SNPs for PH, thirty SNPs for EH, and collectively identified one gene associated with PH and EH [27]. An association population including ten RIL populations was analyzed; 38 SNPs and 43 SNPs associated with plant height and ear height were identified, respectively [28]. Yang et al. conducted 513 maize inbred lines with tropical, subtropical, and temperate characteristics as materials for the GWAS analysis of traits, such as maize plant height, and detected an SNP locus located on chromosome three [29]. In this study, exploiting 226 inbred lines as an association population to conduct a GWAS analysis, it was found that six QTNs were detected in three environments (Table 3). With the aid of the maize GDB database, two candidate genes were identified to associate with significant markers. *Zm00001d021574* encodes the laz1-1 protein and is a member of the *ZmLAZ1* gene family, which acts as transmembrane organic solute transporters and plays a role in a variety of pathways and organs, tissues, or organelles at different developmental stages [30]. ZmLAZ1-4 is a novel zinc transporter that can transport zinc and regulate zinc homeostasis via the negative regulation of the ZmBES1/BZR1-11 transcription factor [31]. In addition, *Zm00001d044730* encodes the calcium-binding EF-hand protein and exhibited a certain level of expression in salt tolerance and permeability of different crops. GsCML27 acts as a Ca2+-binding EF-hand protein in the plant response to bicarbonate, salt, and osmotic stress [32]. *Osccd1* encodes a novel small calcium-binding protein with a C-terminal centrin-like domain. The permeability and salt tolerance of rice seedlings may be positively regulated by influencing *DREB2B* and its downstream genes [33]. *TaCab1* is involved in basal tolerance to biological and abiotic stresses through the SA signaling pathway, and its expression is induced via high salinity and low temperature [34].

QTL analysis is a common method used for the study of quantitative traits by plant breeders [35,36,37]. To construct a genetic map by using linkage markers, many QTL distributed on different chromosomes were detected [35,36,37]. In this study, a high-quality genetic map was constructed with 62,272 markers. In the linkage analysis, 32 significant QTL were detected (Table 4). Abiskar Gyawali et al. identified 37 genomic regions and 25 significant SNPs for plant height by using bulk segregant analysis (BSA) [38]. Teng et al. [39]. identified a quantitative trait locus of qPH3.1 related to PH and one candidate gene of ZmGA3ox2 [39]. Yin et al. [40]. used the NAM population as a material, identified a high-confidence QTL of PH on chromosome one, and explored the candidate gene of *Zm00001d031938* [40]. Li et al. used RILs in five environments to identify one QTL of qPH.A-1.3 [41]. Currently, 219 reported QTL for plant height, 26 QTL for ear height, and 6 QTL for leaf number were searched in the Gramene database. Compared with these reported QTL, we found four QTL (qPH4-2, qEH5-2, qLN1-2, and qLN8-1) with overlapping regions, which can prove that these significant loci are hot intervals for plant architecture trait research. Tanksley concluded that the rate of phenotypic variation for the major genes needs to be greater than 10% [42]. The phenotypic variation for the four QTL in our study was less than 10%. Therefore, in the present study, they were minor gene loci. It is worthwhile to note that a major QTL located on chromosome seven explained more than 10% of the phenotypic variance in our study. However, we did not find QTL for leaf numbers with overlapping intervals in the Gramene database. This will be an important instruction for improving plant architecture traits. Of course, this needs to be verified through more experiments. We will follow up with multi-year experiments to complement the data with the purpose of acquiring more repetitive loci.

The combined analysis of three approaches, GWAS, QTL mapping, and transcriptome analysis helped us to rapidly identify the genetic intervals and mine candidate genes [16,43,44]. Of the two markers identified using the GWAS analysis, SYN34204 and the major gene locus, qLN7-2, identified via the linkage analysis, were both on chromosome seven and were separated by a genetic distance of 15.1cM. The interval of 7:145591031-7:161578861 may be a significant genetic region for plant architecture. In addition, SYN34204 was found to associate with EH and LN in Zhangye, and in different environments (Zhangye and Guiyang), it was detected in association with LN. SYN37324 was found to associate with EH and LN in Guiyang. These results suggest a possible relatively strong correlation between EH and LN. A functional analysis was also carried out for the two identified candidate genes, *Zm00001d021574* and *Zm00001d044730.* The two candidate genes have different functional properties.

Gene function analyses and gene involvement pathways can be instrumental in improving plant architecture. For example, Li et al. proposed that the overexpression of *ZmPIN1a* formed developed roots with long primary roots and dense lateral roots, thus reducing plant height, internode length, and panicle height, which provided the possibility to improve plant architecture and yield [45]. *ZmWRKY114* regulates plant height through the GA signaling pathway [46]. *ZmTE1* is a key regulator of plant height and maintains the orderly formation of the internode meristem and the rapid elongation of cells [47]. The overexpression of *ZmPHYC2* can moderately reduce plant height and ear height [48]. In summary, we found that a single gene mutation of *ZmTE1* or the overexpression of *ZmPHYC2* may affect plant architecture through different pathways. In our study, it was speculated that *Zm00001d021574*, located on the plasma membrane, may interact with Atg8 and participate in the autophagy pathway, but how they combine to affect plant architecture needs to be studied in depth in the future. *Zm00001d044730* probably interacted with *ms26*, which is located on chloroplasts and participates in cutin, suberin, and wax biosynthesis. *MS26/CYP704B* is required for anther and pollen wall development in bread wheat [49]. The targeted mutagenesis of a cytochrome P450-like gene (*MS26*) produced male-sterile maize plants and resulted in vegetative sterility in sorghum and rice [50,51]. Interactions with male sterility genes may be a new pathway for plant architecture regulation, and more experiments will follow to verify this possibility. In addition, the factors of plant architecture also include the leaf angle. The examination of related phenotypic data and the study of leaf angle were not carried out in this study, and more experimental data will be added to illustrate our results in the following period as well. It was speculated that photosynthesis is involved in plant architecture regulation. These speculations are all from different databases, and verification studies need to be performed. However, these results provide some theoretical support for plant architecture improvement.

## 4. Materials and Methods

### 4.1. Plant Materials and Field Experiment

In this study, one natural population with 226 inbred lines as association panel materials derived from the Suwan population of 98 accessions and temperate resources of 128 accessions was planted and phenotyped in Sanya (109.5° E, 18.2° N), the Hainan Province, Guiyang (106.71° E, 26.57° N), the Guizhou Province, Zhangye (100.46° E, 38.93° N), and the Gansu Province in 2020. In 2019, F2 generation derived from T32 crossed with Qi319 was planted in Sanya and one F2:3 of 150 family lines as a linkage population was planted and phenotyped in Zhangye and Guiyang in 2020. The association panel materials and F2:3 family lines were selected as the experimental materials. The field experiment of the linkage population was conducted in a randomized block design with two replicates. Each line was planted in a single row 3 m in length with 0.7 m between the adjacent rows and 12 individual plants per row. All field trials of fertilization, irrigation, pest control, and weed management were the same as those of the local field.

### 4.2. Plant Architecture-Related Trait Evaluation and Statistical Analysis

The associated traits of plant architecture in this study, including plant height, ear height, and leaf number, were measured in 2020. After 20 days of flowering, plant height (PH), leaf number (LN), and ear height (EH) were measured for 5 plants in each plot. All phenotypes were performed according to our previously described method [52]. The PH was measured from the soil surface to the tip of the tassel, and the EH was measured from the soil surface to the node of the upper ear. The LN was counted for all the leaves. The phenotypic data were analyzed using the frequency procedure of IBM SPSS statistics 24.0 software. An analysis of variance (ANOVA) was performed using PROC GLM in the SAS 9.0 with the genotype, environments, and interaction between genotype and environment. Then, the broad-sense heritability (H^2^) for plant architecture traits was calculated using ANOVA-based variance components suing the method by Hallauer and Miranda [53]. The phenotypic data for each line in each the environment were averaged and collated in an Excel spreadsheet for the correlation analysis. The correlation coefficient matrix was calculated using the R package “datasets” and “corrplot” and to was visualized in R studio 4.2.2.

### 4.3. DNA Extraction and Genotyping

The DNA extraction and genotyping methods were described in our previous report [54]. The genomic DNA was extracted from the young leaves of each individual plant using the modified CTAB procedure [55]. The DNA quality and GBS assessments were carried out at the Beijing Compass Biotechnology Company according to previously described methods [56]. ApeKI (New England Biolabs, Ipswich, MA, USA) was used for fragmentation, and 152 digested DNA samples, distinguished with 4–8 base barcode adapter indices, were combined and purified using a QIAquick PCR Purification Kit (Qiagen, Valencia, CA, USA). The ligation products from each library were amplified by using a Phusion HighFidelity PCR Kit (New England Biolabs, Ipswich, MA, USA) in 50-lL volumes. DNA fragments between 170 and 350 bp were thus enriched in libraries and prepared for next-generation sequencing with an Illumina HiSeq 2000 sequencer. The raw reads were sorted according to the indices, and the high-quality SNPs between the parents were identified via alignment with the B73 RefGen_v4 sequence (www.maizegdb.org (accessed on 15 December 2023)) using the BWA package [57] and the Genome Analysis Toolkit (GATK). The calling and annotation of the SNPs were accomplished using Samtools 1.9 [58].

### 4.4. Genome-Wide Association Study and QTL Mapping

Based on the minor allele frequency (MAF) >0.05 and deletion rate <20%, 43,252 high-quality SNP markers were selected for the genome-wide association analysis using the MLM model of the Farm CPU package, in which the population structure and pairwise kinship were treated as covariates of TASSEL v5.2.80 software [59]. In fact, the kinship determination, population structure detection, and principal component analysis (PCA) of the association panel had already been completed in our previous research. The LD decay distance was 40.15.

Kb, the interval, was deduced by the peak position ∓(1 × LD distance) [54]. The logarithm of the odds (LOD) score >3.0 was set as a threshold for significance [60]. For the QTL mapping analysis, SNPs were excluded with a minor allele number (MAF) < 0.05. After this, 62,272 high-quality SNP markers were selected to construct a genetic map and linkage analysis. This high-quality marker information was collated in csv format file, in which the first column was the linkage groups on the genetic map, the second column was the genetic distances for each locus on the genetic map, and the third column was the names of the loci, which were then visualized using the R package “LinkageMapView” in R studio 4.2.2. The LOD threshold was set to 3.0 for the QTL detection.

### 4.5. Candidate Gene Function Prediction

In this study, the functional prediction of two candidate genes was performed using the NCBI database (https://www.ncbi.nlm.nih.gov/ (accessed on 15 December 2023)). The introns and exons of the candidate genes were visualized using GSDS tools. The Ref-Expression tool of the ZEAMAP website (http://www.zeamap.com/ (accessed on 15 December 2023)) was used to analyze the spatiotemporal expression patterns. The WoLF PSORT tool (https://wolfpsort.hgc.jp/ (accessed on 15 December 2023)) was used to locate the encoded proteins and the cell locations of the biological functions. Finally, the STRING database (https://cn.string-db.org/ (accessed on 15 December 2023)) was used to predict the gene interactions.

### 4.6. Transcriptome Analysis

The transcriptome analysis method was described in our previous report [61]. T32 and Qi319 were planted at the Sanya and Zhangye sites. The leaves were collected from three replicates of each inbred line at the V9 stage. A total of 42 samples were collected for total RNA extraction. The total RNA was extracted using the TRIzol reagent, and the construction of the cDNA libraries and RNA sequencing were performed using Biomarker Technologies (Beijing, China) with the Illumina HiSeq 2000 platform. The clean reads were mapped to the maize B73 reference genome assembly V4 by using TopHat2 [62]. The gene expression level was estimated by using the fragments per kilobase per million reads (FPKM) value. The differentially expressed genes were obtained by using the R 4.2.2 statistical software package DESeq with Padj < 0.05 and |log2(fold change [FC])| ≥ 1 [63].

## 5. Conclusions

This study showed that QLN7-2 was a dominant QTL associated with plant architecture, tightly linked with 15.1 Mb SNP SYN34204 and significantly associated with plant architecture-related traits. Two candidate genes, *Zm00001d021574* and *Zm00001d044730*, were identified to be related to maize plant architecture. The bioinformatics and transcriptome analyses showed that of these two candidate genes, *Zm00001d021574* may be an important candidate gene for potentially regulating plant architecture traits because of its participation in the autophagy pathway. The further development and functional characterization of the genetic regions in which the two genes are located will help to improve the maize plant architecture.

## Figures and Tables

**Table 1:** Descriptive statistical analysis for maize plant architecture-related traits.

Environment	Trait	Value (cm)	Range ^a^	Skewness	Kurtosis	CV(%) ^b^
Zhangye	PH	214.95 ± 30.05	138.33~310.00	0.269	0.612	14
	EH	95.20 ± 24.35	31.67~171.67	0.615	0.572	26
	LN	15.2 ± 2.04	10.00~21.67	0.37	0.302	13
Guiyang	PH	170.09 ± 29.18	82.33~259.67	−0.008	0.156	17
	EH	56.49 ± 15.04	19.67~94.67	0.226	−0.258	27
	LN	13.41 ± 1.14	11.00~15.00	−0.257	−1.122	8
Sanya	PH	185.16 ± 25.60	123.33~258.00	0.046	−0.11	14
	EH	62.79 ± 13.49	26.67~108.33	0.175	0.13	21
	LN	12.23 ± 1.33	8.33~15.67	0.188	0.249	11

Note: ^a^ The values are in centimeters. ^b^ CV is the ratio of the standard deviation to the mean.

**Table 2:** Analysis of plant architecture variance in the three environments.

Source of Variance	Genotype	Environment	Genotype × Environment	Broad-Sense Heritability H^2^ (%)
PH	350.62 **	523.88 **	593.99 **	62
EH	126.52 **	428.20 **	271.92 **	56
LN	0.65 **	2.25 **	2.17 **	45

Note: ** indicates significant differences at the 0.01 level.

**Table 3:** SNPs associated with plant architecture-related traits.

SNP Name	Chromosome	Position	Candidate Interval	Trait
PZE-101198702	1	246065082	246024932–246105232	PHSY, EHGY
PUT-163a-78121249-4396	3	141912418	141872268–141952568	EHGY, LNGY
SYN21465	4	184629742	184589592–184669892	EHZY, LNZY
PZE-106027247	6	64142485	64102335–64182635	PHGY, EHGY
SYN34204	7	145631181	145591031–145671331	EHZY, LNZY, LNGY
SYN37324	9	1038593	998443–1078743	EHGY, LNGY

**Table 4:** Plant architecture-related QTL detected in different environments.

Trait	QTL	Chr.	Left Marker	Right Marker	Environment	Lod	PVE (%)	Add
PH	qPH3-1	3	S3_187960135	S3_188121526	ZY	4.61	6.57	5.48
	qPH4-1	4	S4_43000415	S4_43093206	GY	3.00	5.19	12.07
	qPH4-2	4	S4_38067503	S4_38893611	ZY	3.14	4.37	−3.41
	qPH8-1	8	S8_121726317	S8_122100797	ZY	3.03	3.64	−3.29
EH	qEH1-1	1	S1_268192037	S1_268327863	ZY	5.51	5.79	14.99
	qEH5-1	5	S5_59950812	S5_60043203	GY	3.00	9.20	14.75
	qEH5-2	5	S5_135586624	S5_136062818	ZY	3.34	5.88	13.52
	qEH9-1	9	S9_13399713	S9_14224384	ZY	3.42	4.13	6.24
	qEH9-2	9	S9_17058222	S9_17191253	ZY	3.88	4.32	5.62
	qEH9-3	9	S9_21938538	S9_22119724	ZY	3.63	4.26	9.18
	qEH9-4	9	S9_62092382	S9_62374416	ZY	3.22	3.94	9.30
	qEH9-5	9	S9_117989798	S9_118700100	ZY	3.53	5.88	−12.50
LN	qLN1-1	1	S1_71148329	S1_71295345	GY	39.76	4.93	−26.78
	qLN1-2	1	S1_121195417	S1_121304991	GY	30.65	4.92	−26.72
	qLN2-1	2	S2_46244778	S2_46383849	GY	27.79	4.92	−26.71
	qLN2-2	2	S2_108284115	S2_108372309	GY	4.31	4.92	−26.70
	qLN3-1	3	S3_5011915	S3_5113035	GY	33.59	4.92	−26.75
	qLN4-1	4	S4_98857060	S4_99140779	GY	30.20	4.92	−26.72
	qLN4-2	4	S4_203027166	S4_203092635	GY	26.17	4.92	−26.73
	qLN4-3	4	S4_242005439	S4_242167358	GY	38.87	4.93	−26.66
	qLN5-1	5	S5_131941861	S5_132084920	GY	39.00	4.92	−26.71
	qLN6-1	6	S6_116958340	S6_117047670	GY	42.2703	4.93	−26.56
	qLN7-1	7	S7_1076898	S7_1130364	GY	44.3195	4.93	−26.67
	qLN7-2	7	S7_160730663	S7_161578861	ZY	4.829	18.11	0.95
	qLN8-1	8	S8_96818124	S8_97337845	GY	34.2739	4.93	−26.67
	qLN8-2	8	S8_138010301	S8_138087002	GY	32.0265	4.93	−26.57
	qLN8-3	8	S8_145998976	S8_146131133	GY	33.7216	4.93	−26.79
	qLN10-1	10	S10_35037696	S10_35186671	GY	3.064	1.66	9.17
	qLN10-2	10	S10_58062903	S10_58090968	GY	37.6121	4.92	−26.77
	qLN10-3	10	S10_69014140	S10_69182216	GY	31.1681	4.93	−26.62
	qLN10-4	10	S10_137044625	S10_137150459	GY	35.7406	4.92	−26.73
	qLN10-5	10	S10_19080057	S10_19080191	ZY	3.9703	5.77	0.70

Note: The positive and negative additive effects indicate alleles from the female parent and male parent, respectively.

**Table 5:** The total genes in the two significantly downregulated DEG enrichment pathways.

NO.	Gene ID	Regulation	Annotation
1	*Zm00001d022445*	Downregulated	Alpha galactosidase A
2	*Zm00001d006682*	Downregulated	Zinc finger, C2H2 type
3	*Zm00001d048783*	Downregulated	Glycosyl hydrolases family 35
4	*Zm00001d041861*	Downregulated	Alpha galactosidase A
5	*Zm00001d004434*	Downregulated	Alpha galactosidase A

## Data Availability

The data presented in this study are available upon request from the corresponding author.

## Supplementary Materials

The following supporting information can be downloaded at: https://www.mdpi.com/article/10.3390/ijms25052694/s1.
