# Supplementary material for: Joint-GWAS, Linkage Mapping, and Transcriptome Analysis to Reveal the Genetic Basis of Plant Architecture-Related Traits in Maize
Source: Int J Mol Sci. 2024 Feb 26;25(5):2694. doi: 10.3390/ijms25052694 (PMC10931974; doi:10.3390/ijms25052694)
Supplement: Supplementary file 1 [file ijms-25-02694-s001.zip › ijms-2811736-supplementary.pdf]

International Journal of Molecular Sciences

MDPI

Supplementary Materials

Table S1. Description of SNPs significantly associated with maize plant architecture

Trait	Environment	SNP name	Chromosome	Position	-log10(P)	R2(%)
		SYN35346	1	2724801	4.70	11.25
		SYN6318	1	6206622	4.62	3.25
		PZE-101213253	1	262359150	5.93	1.75
		PZE-101160778	1	202855995	5.07	19.21
		PUT-163a-78122363-4402	1	284147312	4.48	1.66
		PZE-102133103	2	183597022	4.26	3.77
		PZE-102027520	2	12799478	5.09	3.33
		PZE-102121473	2	164391609	5.11	3.19
PH	Zhangye	PZE-103128516	3	183511214	6.99	10.63
		PZE-103057113	3	69933153	8.27	25.87
		SYN6704	3	214837865	6.52	10.73
		PZE-104112214	4	193127992	4.82	18.99
		PHM14235.14	4	3069054	5.66	3.68
		PZE-104004875	4	3547938	4.22	2.67
		SYN11697	4	9730261	5.87	8.25
		ZM013874-0376	6	82077772	6.67	9.39
		SYN31213	7	109722314	5.46	4.03

	PZE-108002410	8	2524458	7.07	5.54
	PZE-108040089	8	63389712	4.26	3.90
	PZE-109027579	9	27901818	13.08	11.55
	SYN26907	9	142982585	7.82	6.71
	SYN9733	10	81929828	6.23	4.50
	PZE-101192491	1	238400676	5.15	9.19
Guiyang	ZM013346-0450	4	237683350	5.52	17.53
	PZE-106027247	6	64142485	5.95	11.93
	PZE-101102168	1	99207957	7.20	16.00
	PZE-101102244	1	99683790	9.27	15.77
	PZE-101198702	1	246065082	6.97	16.78
	SYN31829	1	262344483	5.45	6.06
	SYN434	1	288440329	7.77	12.98
	SYN19476	2	32319562	5.81	4.4
Sanya	SYN801	3	60244907	5.80	4.51
	SYN31292	3	163898354	8.68	10.55
	PZE-104025181	4	29205220	5.73	6.57
	PZE-104092741	4	167834280	5.52	13.5
	SYN5723	5	655460	5.51	6.5
	SYN37836	5	9865064	4.85	8.41
	PZE-106064449	6	116335450	7.2	16

		SYN28847	10	47067413	4.82	6.42
		PZE-110066900	10	123022997	4.96	5.93
	Zhangye	SYN20042	1	284052211	5.88	36.18
		SYN2041	1	84622747	3.80	2.93
		SYN14016	1	295083826	8.36	9.19
		PZE-101064527	1	47595480	5.51	6.33
		SYN16785	2	12097490	3.88	6.01
		PZE-102109004	2	140255184	4.07	8.04
		PZE-104051887	4	89327390	4.71	3.31
		SYN21465	4	184629742	7.24	11.60
		PZE-104050891	4	87110052	4.41	7.83
EH		PZE-105146867	5	198973141	3.85	3.51
		SYN34204	7	145631181	4.53	3.61
		PZE-107062095	7	112910593	4.53	6.84
		SYN20446	8	166991645	7.45	10.15
		PZE-108079155	8	133836445	3.97	3.23
		PZE-109060918	9	99719655	6.51	7.01
		PZE-110072208	10	128385969	5.39	5.51
		PZE-110040909	10	77905176	4.52	5.89
		PZE-110076169	10	131863026	3.79	8.96
	Guiyang	PZE-101198702	1	246065082	5.32	12.99

	PZE-101157937	1	199991908	4.76	4.21
	PZE-102097892	2	113905259	5.26	6.15
	SYN13768	3	27198816	4.54	4.61
	PUT-163a-78121249-4396	3	141912418	4.22	4.96
	PZE-104051841	4	89297959	6.84	5.51
	PZE-104067920	4	134117096	3.77	4.28
	PZE-105106149	5	160882618	5.45	5.40
	SYN27136	5	13993759	4.46	3.55
	PZE-105112344	5	168635990	3.92	1.65
	PZE-106027247	6	64142485	7.68	9.69
	PZE-107018058	7	15349014	4.24	2.65
	PZE-107041844	7	71317652	4.94	9.08
	SYN37324	9	1038593	5.09	5.92
	PZA01313.2	10	3598262	8.57	16.19
	PZE-110051214	10	96268908	4.38	4.58
Sanya	PZE-103026453	3	19255311	5.19	4.57
	SYN17581	3	174742209	5.40	9.52
	SYN2182	4	159047308	6.10	14.41
	PZE-104086560	4	160131973	5.17	7.57
	PZE-104132625	4	221598254	5.17	9.64
	PZE-107050482	7	94446621	5.90	16.81

		PZE-109021971	9	22099758	5.35	6.71
	Zhangye	PZE-101161688	1	204081525	4.32	6.56
		PZB01979.2	1	224421551	6.12	7.85
		PZE-101124789	1	157170910	5.61	5.74
		PZE-102170743	2	213992430	4.55	10.83
		SYN4868	3	227812313	4.86	7.92
		PZE-103032119	3	24390862	4.14	3.71
		SYN21465	4	184629742	8.75	17.38
		SYN24008	4	241827929	4.46	6.16
		SYNGENTA10922	6	156740334	5.16	6.06
LN		SYN34204	7	145631181	5.07	8.18
		SYN32385	7	129793852	5.24	5.41
		PZA03543.3	8	107830603	5.21	5.59
		SYN13997	10	10158769	3.96	8.31
		PZE-110026998	10	44812890	3.89	4.40
		PZE-110010880	10	9080966	4.61	4.35
		PZE-110038968	10	74737641	4.28	4.91
	Guiyang	PZE-101197298	1	244831238	6.35	7.93
		PZE-101005806	1	4452918	4.26	6.40
		SYNGENTA16007	1	60158566	5.03	6.63
		PUT-163a-78121249-4396	3	141912418	4.48	5.34

	PZE-104053856	4	93692770	6.58	9.03
	PZE-104132437	4	221197797	5.11	5.75
	SYN33594	5	162869078	8.05	15.80
	PZE-106074560	6	130227960	5.92	8.74
	SYN34204	7	145631181	3.89	2.21
	PZE-107025209	7	29607900	5.23	8.82
	SYN7980	8	137481079	5.75	15.21
	SYN17572	8	21183591	5.49	5.21
	SYN37324	9	1038593	5.05	4.57
	PZE-109029505	9	32329732	3.72	5.49
	PZE-110063766	10	118814841	4.43	9.46
	PZE-110026005	10	42521152	6.13	6.93
Sanya	SYN3043	3	148155393	5.23	3.75
	PZE-105096177	5	140706333	7.38	5.29
	PZE-105068275	5	70167652	4.92	4.84
	ZM013186-0134	9	145603981	5.52	17.53
	PZE-110033620	10	63293673	5.06	19.21
	PZE-110052027	10	98138896	7.02	9.48
	SYN12162	10	43110396	7.54	12.22

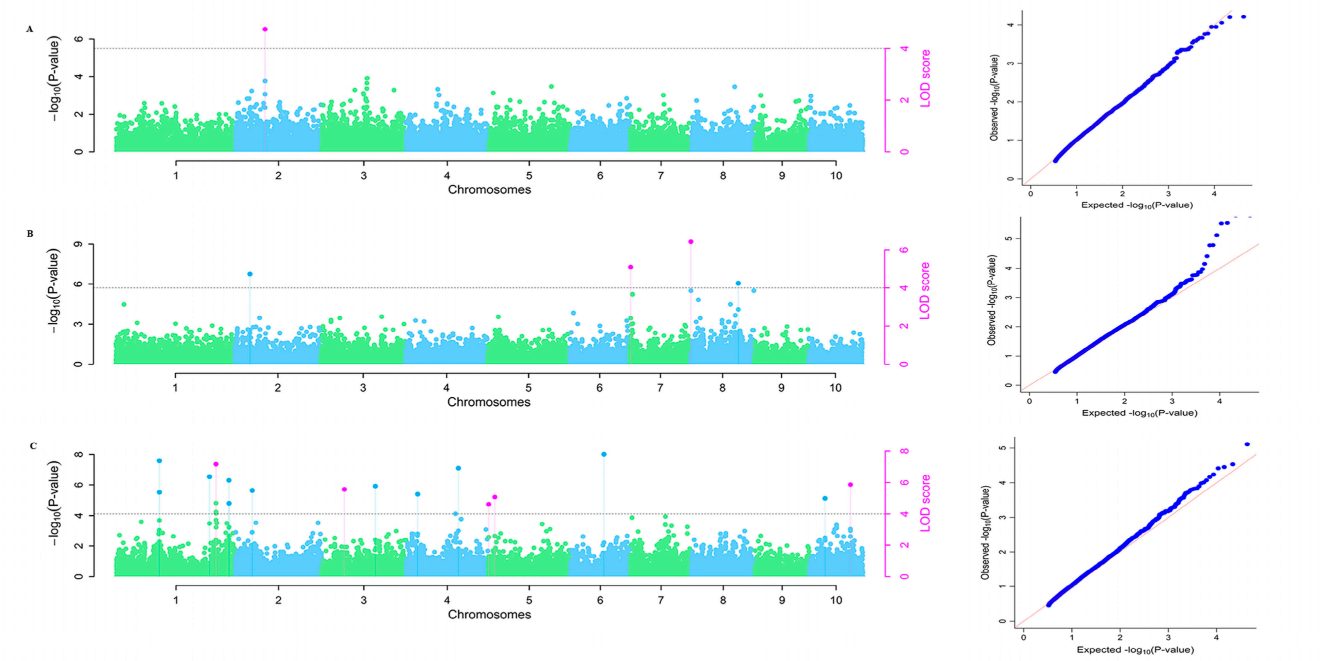

Figure S1. Manhattan and QQ plots for maize ear height under different environments. A was Guiyang, B was Zhangye , C was Sanya. a significance LOD score level was more than 3.0 ; Left plots were Manhattan , right plots were quantile−quantile (Q−Q) .

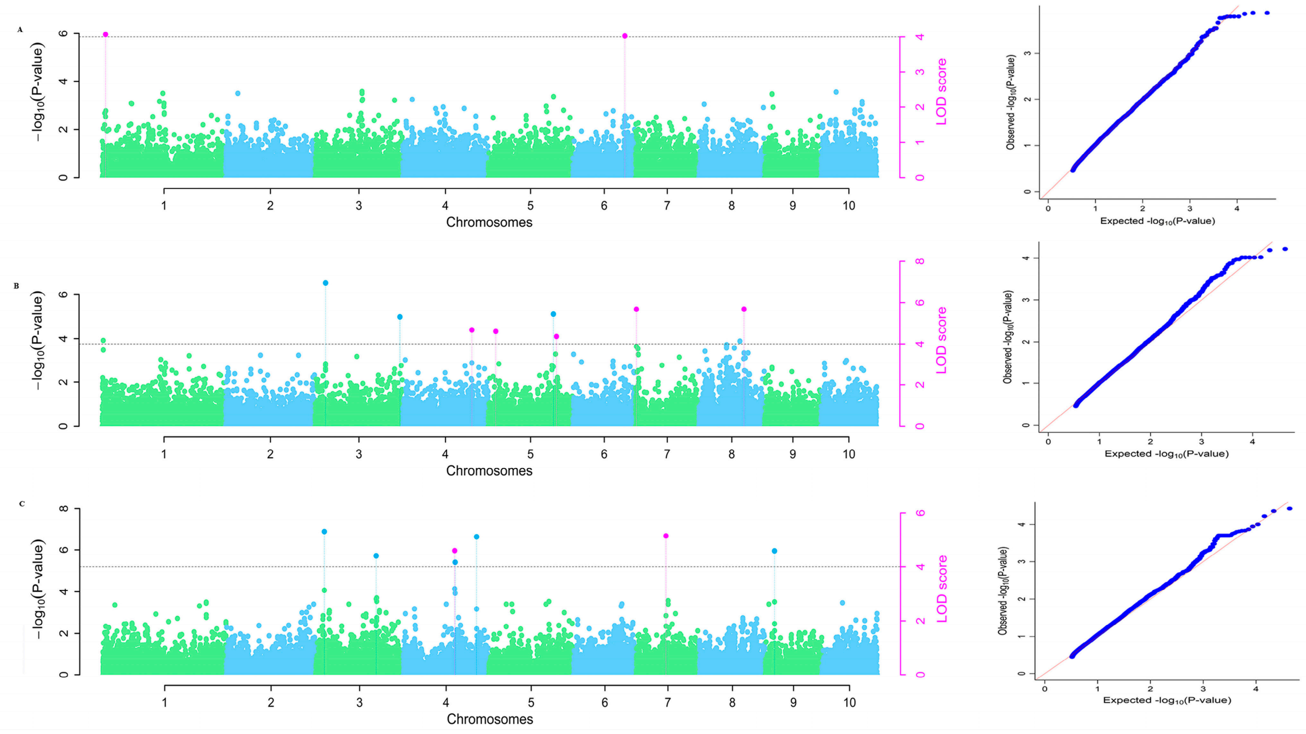

Figure S2. Manhattan and Q−Q plots for maize leaf number under different environments. A was Guiyang, B was Zhangye , C was Sanya. a significance LOD score level was more than 3.0; Left plots were Manhattan, right plots were quantile−quantile (Q−Q).
